# Cerebral Air Embolism Following Endoscopic Retrograde Cholangiopancreatography Stent Removal: A Rare and Fatal Complication

**DOI:** 10.1155/cria/6740961

**Published:** 2026-11-06

**Authors:** Bavani Senthuran, A. Arulmurali, Anuratha Kajendran, Thishanthini Vijayacumar, Shathana Paramanathan

**Affiliations:** ^1^ Department of Surgery University of Jaffna Jaffna Sri Lanka jfn.ac.lk; ^2^ Teaching Hospital Jaffna Jaffna Sri Lanka thjaffna.lk

**Keywords:** cerebral air embolism, endoscopic retrograde cholangiopancreatography, hyperbaric oxygenation, target-controlled infusion

## Abstract

**Background:**

Endoscopic retrograde cholangiopancreatography (ERCP) is a generally performed procedure for biliary pathology. Although usually safe, air embolism is an exceptionally rare but potentially fatal complication.

**Case Presentation:**

We report the case of a 79‐year‐old female (ASA II) with a history of dyslipidemia and obesity, who presented for elective ERCP and biliary stent removal. She had previously undergone ERCP and open cholecystectomy for choledocholithiasis. During this time, sedation was administered by using fentanyl, midazolam, and propofol via target‐controlled infusion. The procedure was initially uneventful; however, at its conclusion, the patient developed sudden desaturation, bradycardia, and a reduced level of consciousness. Computed tomography (CT) imaging revealed multiple cerebral air emboli. Despite intensive care management and ventilatory support, the patient deteriorated and died 2 days later.

**Conclusion:**

This case highlights the importance of maintaining vigilance for air embolism, even in seemingly straightforward ERCP procedures, especially in elderly patients. Early recognition and immediate management are vital to improving outcomes.

## 1. Introduction

Endoscopic retrograde cholangiopancreatography (ERCP) is a cornerstone in the diagnosis and management of biliary and pancreatic disorders. Although complications such as pancreatitis, bleeding, and perforation are well documented, cardiopulmonary adverse events should not be considered negligible, accounting for 4%–16% of ERCP‐related adverse events [1]. Cerebral air embolism (CAE) remains exceedingly rare, often underrecognized, and the estimated incidence of post‐ERCP is around 0.0033% [2]. Despite its rarity, CAE is associated with high morbidity and mortality. We present a fatal case of CAE following ERCP stent removal in an elderly patient to underscore the importance of awareness, early diagnosis, and timely intervention.

## 2. Case Presentation

A 79‐year‐old woman with a background of dyslipidemia, obesity (BMI 30), and previous ERCP with open cholecystectomy for choledocholithiasis was admitted for elective ERCP and biliary stent removal. Preprocedural vital signs were stable: blood pressure 145/84 mmHg, heart rate 88 beats per minute, and oxygen saturation 98% on room air. The patient was conscious, cooperative, and oriented. Electrocardiography showed normal sinus rhythm. Furthermore, no abnormalities of the papilla were reported on the previous preprocedural biliary computed tomography (CT) scan.

She underwent ERCP in a semiprone position under conscious sedation with intravenous fentanyl 50 μg, midazolam 2 mg, and propofol administered via target‐controlled infusion (effect‐site concentration of 2 mcg/mL). Additional medications included intravenous co‐amoxiclav 1.2 g and 500 mL of 0.9% normal saline. Hyoscine was not administered.

During the ERCP procedure, the previously placed biliary stent was removed using retrieval forceps without the use of a guidewire. A sphincterotomy had been performed at the time of the original stent insertion; however, no extension of the previous sphincterotomy was undertaken during the most recent ERCP. There was no evidence of concurrent inflammation during the most recent ERCP. Following stent removal, contrast medium was manually injected to visualize the biliary tree.

The procedure proceeded without complications until its conclusion, when the patient suddenly developed oxygen desaturation to 85%, bradycardia, and loss of consciousness. Neurological assessment revealed a Glasgow Coma Scale (GCS) of 4/15. This clinical event occurred immediately following the manual injection of contrast medium at the end of the stent removal procedure. Cardiopulmonary resuscitation was not required, as the patient did not experience cardiac arrest. However, endotracheal intubation was performed for airway protection, followed by initiation of volume‐controlled mechanical ventilation. An orogastric tube was inserted, and the patient was transferred to the surgical intensive care unit (ICU).

Immediately following admission to the ICU, a bedside transthoracic echocardiogram was performed and demonstrated no significant abnormalities. A noncontrast CT (NCCT) of the brain performed post‐ERCP revealed multiple areas of CAE, without evidence of infarction or hemorrhage (Figure 1). Neurosurgical consultation was obtained; conservative management with ventilatory support was advised. The patient clinically deteriorated over the next 12 hours and brainstem reflexes were absent. Despite ICU care, serial imaging on Postprocedure Day 2 showed progression of the air embolism, diffuse cerebral edema, and mass effect, along with developing tonsillar herniation (Figure 2). The patient unfortunately died shortly thereafter.

**FIGURE 1:**
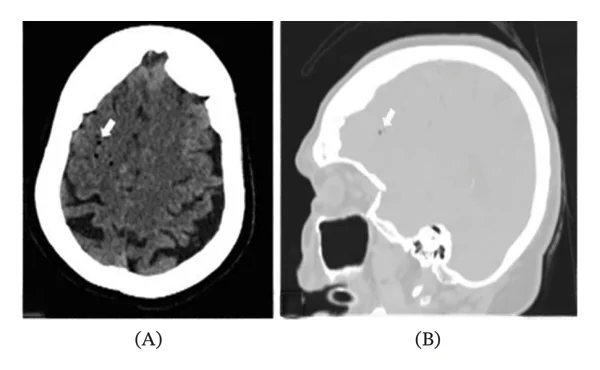
NCCT brain images of Day 1 show intraparenchymal air locules (white arrowheads) in the right cerebral hemisphere. (A) Brain window axial. (B) Lung window sagittal.

**FIGURE 2:**
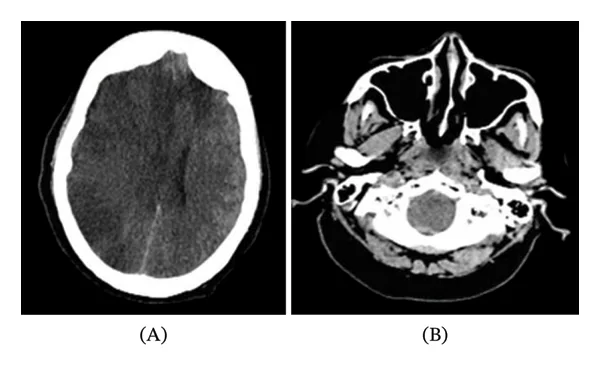
NCCT brain axial images of Day 2 show cerebral edema (A) with herniation (B).

## 3. Discussion

CAE is an infrequent but catastrophic complication of ERCP, often resulting from inadvertent insufflation of air into the biliary system, which then enters the venous circulation or crosses into the arterial system via a right‐to‐left shunt. While the exact mechanism in this case remains uncertain, contributing factors may include mucosal disruption, prior surgical interventions, or a preexisting biliary‐venous communication [3].

A large volume (∼5 mL/kg) or rapid entry of gas migrating to the pulmonary circulation places strain on the right ventricle. As the pulmonary arterial pressure increases, the left ventricular preload decreases. This situation results in diminished cardiac output that leads to cardiovascular collapse and cerebral hypoperfusion [4]. Venous gas embolism may migrate to the systemic circulation through a shunt such as a patent foramen ovale. However, it has been reported that a large volume of venous gas can enter the arterial circulation through transpulmonary passage of air, without cardiac defects or shunt. The air of the venous circulation enters the arterial systemic circulation and obstructs the end artery, which is a paradoxical embolism. It blocks terminal arterioles as the smaller bubbles cannot continue pass the decreasing diameter of the lumen. Ultimately, the result of this process is brain tissue ischemia in the affected vascular territories [5].

The aspiration of intracardiac air through a central venous or pulmonary artery catheter has been described as a potentially life‐saving therapeutic measure in venous air embolism. Nevertheless, its efficacy is highly dependent on timely diagnosis and rapid catheter placement [6]. In the present case, the potential impact of such an intervention on the patient’s outcome cannot be established and remains speculative.

Precordial Doppler ultrasonography is a highly sensitive method for detecting venous air embolism and may identify embolic events before the development of significant hemodynamic deterioration [7]. Previous studies have demonstrated that Doppler monitoring can detect small amounts of intravascular air and may facilitate earlier recognition and intervention [8]. However, its routine use during ERCP is uncommon, given the rarity of clinically significant air embolism and the practical challenges of positioning and maintaining the Doppler probe during procedures, particularly in the prone position. In our institution, precordial Doppler monitoring was not available. Therefore, this modality was not used in the present case.

Using air instead of CO_2_ increases the risk of CAE, a potentially fatal event due to air bubbles blocking brain vessels. CO_2_ is used because it is more soluble, less likely to persist in circulation, and therefore much safer [5].

Although the procedure was brief and technically uncomplicated, the patient’s advanced age and surgical history may have predisposed her to this event. The absence of hyoscine reduces the likelihood of vagally mediated bradycardia as a primary cause of the acute deterioration.

Management of CAE includes immediate high‐flow oxygen, cardiovascular support, and hyperbaric oxygen therapy when available [3]. Unfortunately, once embolism reaches the cerebral circulation, outcomes are often poor, particularly in elderly or medically fragile patients.

This case highlights the need for high clinical suspicion in the setting of sudden cardiopulmonary or neurological changes during or immediately after ERCP.

## 4. Conclusion

CAE, though rare, should be included in the differential diagnosis of any sudden clinical deterioration during or following ERCP. This case emphasizes the importance of procedural vigilance, awareness of risk factors, and the necessity of prompt imaging and supportive care. Early recognition and intervention may offer the only opportunity to mitigate this life‐threatening complication.

## Funding

No funding was received for this study.

## Consent

Written informed consent was obtained from the patient’s mother for publication of this case report and any accompanying images.

## Conflicts of Interest

The authors declare no conflicts of interest.

## Data Availability

The data that support the findings of this study are available on request from the corresponding author. The data are not publicly available due to privacy or ethical restrictions.
